# Functional Electrical Stimulation Combined With Reactive Balance Training for Individuals With Incomplete Spinal Cord Injury: A Randomised Clinical Trial

**DOI:** 10.1177/15459683261454942

**Published:** 2026-06-15

**Authors:** Matthew G. Heffernan, Katherine Chan, Jonguk Lee, Lovisa Cheung, Elizabeth L. Inness, Kei Masani, Kristin E. Musselman

**Affiliations:** 1 Rehabilitation Sciences Institute, Temerty Faculty of Medicine, University of Toronto, ON, Canada; 2 KITE Research Institute, Toronto Rehabilitation Institute, University Health Network, ON, Canada; 3 Institute of Biomedical Engineering, University of Toronto, ON, Canada; 4 Department of Physical Therapy, Temerty Faculty of Medicine, University of Toronto, ON, Canada

**Keywords:** spinal cord injury, electric stimulation, balance recovery, postural control, postural balance, accidental falls

## Abstract

**Introduction:**

Individuals with motor incomplete spinal cord injury (iSCI) have difficulty eliciting reactive steps when balance is lost, increasing fall risk. Functional electrical stimulation (FES) applied to the common fibular nerve elicits a flexor withdrawal response, potentially assisting reactive stepping. Combining reactive balance training with FES (Reactive Balance Training [RBT] + FES) may enhance reactive balance control. This study compared the efficacy of RBT + FES to RBT alone in individuals with chronic, motor iSCI.

**Methods:**

Twenty-two individuals (63.9 ± 14.8 years; 3.8 ± 2.8 years post-injury) were randomly assigned to RBT + FES or RBT alone. They completed 18 1-hour training sessions over 6 weeks. Outcome measures were assessed pre-, immediately post- and 6 months post-intervention, and included the behavioural response of the Lean-and-Release test (single-step, 2-step, ≥3-step, or fall) and clinical measures of balance, balance confidence, fall concern, lower limb strength, and proprioception of the ankle. A 2-way mixed analysis of variance assessed group differences over time. Fall incidence was monitored via an online survey for 6 months following training.

**Results:**

Significant improvements over time were observed for single-step and fall responses during the Lean-and-Release test, and on all clinical outcomes (*P* ≤ .01) with the exception of ankle proprioception. No significant group or group × time interactions were found, suggesting similar improvements between groups. There were no significant between-group differences in fall tracking data.

**Discussion:**

Both RBT + FES and RBT alone led to significant improvements in reactive stepping ability, balance control, balance confidence, fall concern, and lower limb strength. However, the FES protocol applied in this study did not provide additional benefit.

## Introduction

Falling is a major concern for people with incomplete spinal cord injury (iSCI).^1^ These individuals have impaired postural control during activities like walking,^2-4^ and the majority will fall at least once per year.^1,5^ Falls can cause serious injury and contribute to a costly increase in hospital admissions.^1,6^ Additionally, a fall can have negative psychosocial consequences, such as a fear of falling that leads to decreased physical activity, community participation, and autonomy.^7-9^

Falls can occur when an individual’s centre of mass (CoM) is not maintained within their base of support (ie, loss of balance). To avoid a fall after an expected or unexpected loss of balance, reactive balance strategies are used to regain control of the CoM position.^10^ One strategy involves executing reactive steps that serve to quickly increase base of support and decelerate the change in CoM position.^10^ However, iSCI causes reduced strength, coordination, and sensory function,^11^ resulting in impaired reactive stepping.^12,13^ For example, in comparison to able-bodied individuals, when people with iSCI experience a simulated forward fall, they are more likely to show a delayed onset of muscle activity, execute multiple steps to recover,^12^ and demonstrate greater variability in reactive step length, width, and height.^13^

Balance interventions for people with iSCI have focussed on balance control during standing or volitional stepping and target an individual’s anticipatory postural control.^14^ Despite the known impairments in reactive stepping^12,13^ and the heightened risk of falling during standing and walking,^5^ only recently has a balance intervention focussing on reactive stepping been investigated for people with iSCI. Reactive Balance Training (RBT) involves repetitive practice of reactive stepping through repeated manual perturbations delivered by a therapist during standing and walking activities that challenge balance control.^15^ RBT is a safe and feasible balance intervention for people with iSCI,^16^ and also an efficacious intervention for other populations with impaired reactive balance control.^17-24^

During RBT, some individuals with iSCI are unable to consistently execute effective reactive steps after a manual perturbation, requiring support to prevent a fall.^16^ For these individuals, functional electrical stimulation (FES) may facilitate a stepping response when balance is lost.^25^ FES can generate appropriately timed muscle contractions to support functional movements like stepping.^26^ When electrical stimulation is applied to the common fibular nerve of an individual with iSCI, a flexor-withdrawal response is initiated, promoting rapid hip and knee flexion and ankle dorsiflexion.^27^ Further, the timing of this response can be controlled with pressure sensors in the shoes such that the FES occurs in close temporal proximity to a perturbation (ie, unweighting of a leg in an attempt to take a reactive step releases pressure on the sensor, activating FES).^28-30^ Hence, this FES application could be used during RBT to create an orthotic effect (ie, immediate assistive effect), whereby individuals with iSCI consistently execute reactive stepping after a manual perturbation, enabling the repetitive motor practice needed for motor learning. Repetitive exposure to FES is also known to improve muscle strength^31,32^ and facilitate neuroplastic change, such as strengthened corticospinal connections,^33,34^ in individuals with iSCI, both mechanisms that could lead to improved reactive stepping ability.

Common fibular nerve FES has been used for gait training in people with iSCI.^27,35^ In a recent case series of 2 individuals with iSCI, combining RBT with FES (RBT + FES), led to clinically meaningful improvements in reactive stepping ability and balance control that persisted post-training without FES.^25^ However, it is unknown whether RBT + FES would lead to greater improvements than those achieved with RBT alone. This knowledge may help clinicians determine whether it is worthwhile to invest in the equipment and set-up time required to incorporate FES into RBT. Hence, the primary aim of this study was to compare the therapeutic effect of RBT + FES to RBT alone on reactive stepping ability as measured by the number of steps taken after a simulated forward fall. The secondary aims included comparing the effects of the 2 interventions on clinical measures of balance, lower limb strength, balance confidence, and concern about falling and ankle proprioception. Additionally, we examined differences between groups in fall data collected during the 6 months post-intervention. We hypothesised that the RBT + FES group would demonstrate greater improvements in reactive stepping ability, measures of balance, lower limb strength, balance confidence, concern about falling, and proprioception compared to those who received RBT alone. We also hypothesised that the RBT + FES group would exhibit a lower fall incidence, fewer total falls, and a longer time to first fall in the 6 months post-intervention.

## Methods

### Study Design

A single-site, assessor-blinded randomised clinical trial (ClinicalTrials.gov identifier NCT04881565) was conducted at the Lyndhurst Centre, University Health Network (UHN). Approval for the study was granted by UHN’s Research Ethics Board (study ID: 21-5210). The manuscript was prepared under the CONSORT 2010 checklist for randomised clinical trials.

### Participants

Individuals with iSCI from the Lyndhurst Centre’s Central Recruitment Database^36^ were contacted to participate. Participants were deemed eligible if they were ≥18 years old; had a traumatic or non-progressive, non-traumatic motor iSCI (ie, American Spinal Injury Association Impairment Scale C or D) diagnosed more than a year before study enrollment; needed physical assistance, a gait aid, or a brace to ambulate 10 meters (ie, Walking Index for SCI II rating of 6-19)^37^; were able to stand for >30 seconds without upper limb support or assistance (ie, scored ≥2/4 on the Standing Unsupported item from the Berg Balance Scale)^38^; and could attend 3 training sessions per week for 6 weeks. Individuals were excluded from participation if they had contraindications to FES (eg, implanted electronic device, recent radiation, active deep vein thrombosis, pregnancy), other conditions affecting balance (eg, vestibular disorder, brain injury), a pressure injury (>grade 2) on the pelvis, trunk, or foot where the safety harness or pressure sensor were placed, a history of lower limb fragility fracture, or severe spasticity or contractures in the lower limbs that prevented the hips or knees from attaining a neutral (0^o^ of extension) position in standing. An in-person screening assessment was conducted to ensure a muscle contraction could be achieved with surface FES in at least 1 tibialis anterior muscle. Similar studies conducted at the Lyndhurst Centre have experienced difficulty in recruiting men^16,39^; hence, men were purposively recruited to participate in this study.

### Outcome Measures

Assessments were conducted at baseline (BL), post-training (PT), and 6-month follow-up (6MFU). Each assessment included the Lean-and-Release test, which assesses reactive stepping ability after a simulated forward fall,^12^ administered by 3 researchers. Participants stood on a platform wearing a safety harness that was secured overhead. A cable instrumented with a force transducer and release mechanism was fastened to the safety harness at waist height. Participants were instructed to lean forward until the cable supported 8% to 12% of their body weight.^40^ The cable was then released at a random time within a 30-second window to initiate an anterior fall. Participants completed up to 10 trials, with an additional 3 false trials (ie, no release) included randomly throughout. Participants were instructed to recover balance in as few steps as possible. For each trial, the number of reactive steps taken or the need for assistance from the harness/researcher (ie, fall response) were recorded.

The primary outcome measure was the behavioural response observed during the Lean-and-Release test. Specifically, the proportion of trials classified as a single, 2-step, ≥3 step, or fall response were compared between groups over time. Under similar conditions, able-bodied individuals typically recover with a single step.^40^ However, in a previous study, 45% of able-bodied adults who completed the Lean-and-Release test took additional steps.^12^ Given that a second reactive step can be a natural response in a forward lean, as it restores a symmetrical stance and stability, we defined a successful recovery as a single or 2-step behavioural response.^41^ Previous work has shown that a change of 0.17 in the proportion of behavioural response during the Lean-and-Release test demonstrates change beyond the standard error of the measure (SEM).^42^

The secondary outcome measures administered by a single physical therapist (LC) blind to group allocation included assessments of balance (Berg Balance Scale [BBS]) and reactive postural control subscale of the mini-Balance Evaluation Systems Test (mini-BESTest), strength, proprioception, and self-reported balance confidence and fall concern. The valid and reliable BBS assesses 14 sitting and standing tasks on an ordinal scale, with a maximum score of 56.^38,43^ The minimal detectable change (MDC) is 4.4 points.^44^ The reactive postural control subscale of the mini-BESTest consists of 3 items with a total of 6 points. This subscale has high reliability and validity in chronic iSCI.^45-47^ A 1-point change in the reactive subscale was considered clinically significant, indicating improved reactive stepping in at least 1 direction.

Lower limb weakness contributes to fall risk in iSCI and FES has been shown to improve muscle strength for these individuals.^5,48^ Therefore, isometric strength was measured through manual muscle testing (MMT) of the hip (extensors, flexors, abductors, and adductors), knee (extensors and flexors), ankle (plantarflexors and dorsiflexors), and long toe (extensors). MMT scores range from 0 to 5, with muscles scoring ≥4 having sufficient strength to also be assessed using hand-held dynamometry, a valid method for assessing strength in SCI that has greater sensitivity to detect changes compared to MMT alone.^49,50^ Three peak force values were collected per muscle group, with the mean value normalised by body weight. Testing positions were standardised, following the guidelines of the International Standards for Neurological Classification of SCI^51^ as appropriate.

Proprioception was evaluated using a bilateral ankle motion discrimination task.^52^ Participants, in a supine position with eyes closed, identified whether they could detect up or down movement of the ankle as the blinded assessor moved the ankle 10 degrees into dorsiflexion or plantarflexion, with six movements performed per ankle.^53^ Scoring was based on the number of correct responses with a maximum total score of 12. This test has demonstrated a relationship between standing balance and proprioceptive function in iSCI^54^ which can potentially improve with movement training like RBT.^55^

The Activities-specific Balance Confidence (ABC) Scale assessed self-efficacy in maintaining balance during standing and walking tasks, with ratings from 0% to 100%, averaged for a total score.^56,57^ The MDC of the ABC Scale for chronic iSCI is 14.9%.^57^ A personalised ABC version allowed participants to select 5 personally relevant tasks, generating a separate mean score. There was considerable variety in the personally relevant tasks identified by participants (see Supplemental File); however, the most commonly identified tasks involved walking under varying environmental and task conditions (ie, gait adaptability), standing activities, and tasks related to sport participation. The Falls Efficacy Scale-International (FES-I) measured concern about falling during physical and social activities, using a 16-item scale rated from “not concerned” to “very concerned.”^58^ The FES-I’s MDC for iSCI is 12.6 points.^16^

Fall monitoring occurred for 6 months post-training, the recommended period for community-dwelling individuals with SCI.^1^ Falls, defined as an event where an individual comes to rest inadvertently on a lower level,^59^ were documented via a survey completed within 24 hours post-fall. Every 3 weeks, participants received an email or phone reminder to ensure survey completion. This method has been effective in tracking falls in SCI populations.^16,60^

### Interventions

Following BL assessment, participants were randomly assigned to the RBT + FES or control group using a computer-generated, blocked randomisation with block sizes of 2 or 4 (KEM). A sequential matched randomisation technique^61^ was used to assign participants to blocks, with matching based on Lower Extremity Motor Score (±3 points).^62^ Participants in each group completed 18 1-hour training sessions over 6 weeks. Training was delivered by a physical therapist (KEM) or kinesiologist (MGH and KC). Before each session, participants donned a safety harness securely attached to an overhead track allowing movement in a 2 m × 4 m space (Prism Medical Ltd., Concord, ON). Approximately 40 to 50 perturbations per session (ie, 1 perturbation per training minute) were applied manually by the therapist in various directions to elicit reactive stepping. The perturbations were pushes and pulls applied at the hip attachment of the safety harness.^16^ The force applied by the therapist was individualised to each participant and each task with the goal of applying sufficient force to elicit a single- or multi-step response. Participants were not given advance notice of the timing or direction of the perturbations. A researcher visually observed and recorded the number of perturbations and behavioural response to each perturbation during training. Perturbations were delivered while participants simultaneously completed standing and walking balance tasks. Task difficulty was tailored to individual ability levels by modifying the environmental (eg, changing the standing/walking surface) and physical (eg, changing the base of support, speed) demands.^15^ After completing a task, participants rated its balance challenge on a visual analog scale from 0 to 10 (0 = very easy, 10 = extremely difficult – would fall without assistance), and researchers tailored the difficulty of the tasks to elicit a rating of 7 or higher.^15,16^

Participants in the RBT + FES group received FES bilaterally with the cathode over the common fibular nerve at the fibular neck and the anode placed on the tibialis anterior (Odstock 2-Channel stimulator, Odstock Medical Ltd., Salisbury, UK) throughout training (Figure 1). Stimulation parameters were tailored such that hip flexion and ankle dorsiflexion/eversion were observed; however, an asymmetric biphasic waveform, frequency of 40 to 50 Hz, amplitude of 60 to 100 mA, and pulse duration of 300 to 350 µs were commonly used. Pulse duration was adjusted during the course of a single training session to account for habituation. The ramp time of the stimulator was set to 0 (ie, no ramp time). Stimulation was triggered using pressure-sensitive foot switches placed under each heel in participants’ shoes. When participants initiated a reactive step by shifting their weight onto the non-stepping limb, pressure was released on the foot switch under the stepping limb and the stimulation was triggered. As the stimulation was delivered throughout the training session, it was also delivered during voluntary stepping. The therapist delivering the perturbations confirmed the stimulation was being triggered at the appropriate time through a light signal on the Odstock 2-Channel stimulator, which was secured to the back of the safety harness. Any adverse events experienced during participation in the study were recorded by researchers using a standardised reporting form.

**Figure 1.**
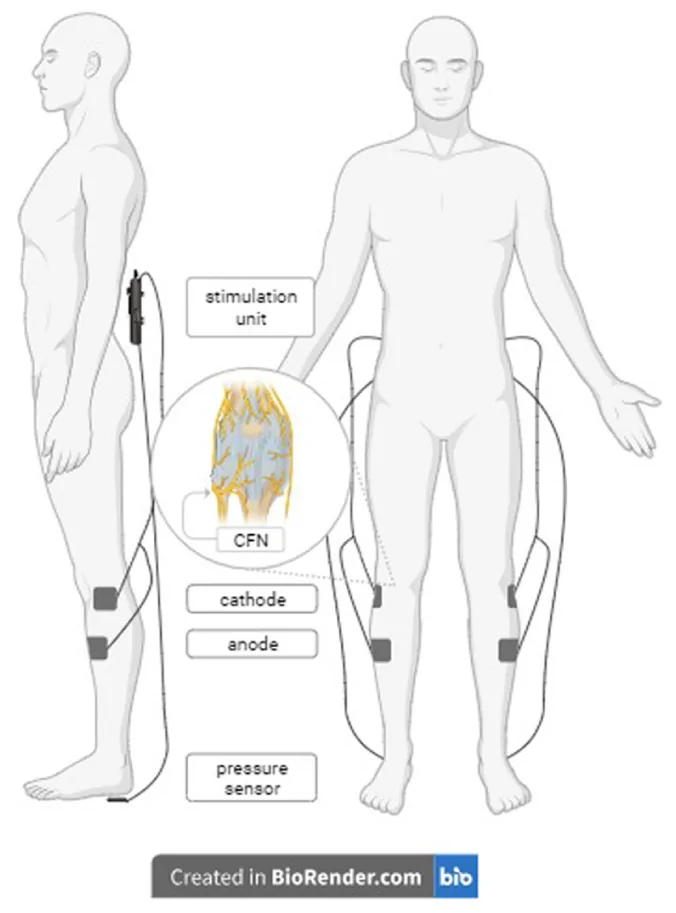
Common fibular nerve (CFN) stimulation for the RBT + FES group. Set up of the Odstock 2-Channel stimulator (Odstock Medical Ltd., Salisbury, UK) for participants receiving RBT + FES to the CFN. The stimulator was secured to the safety harness at the participant’s back. The cathode electrodes were placed over the CFN at the neck of the fibula and the anode electrodes were placed over the tibialis anterior muscles. A pressure-sensitive foot switch was placed in each shoe under the heel.

### Data Analysis

Demographic, injury-related data, and the BL scores of each group were presented as mean ± standard deviation (SD) or frequency counts. The mean number of perturbations per training session were compared between groups using an independent t-test. The changes in outcome measures over time were reported descriptively as mean ± SD or median (interquartile range: IQR), depending on normality, and compared between groups with a 2-way mixed analysis of variance (ANOVA; time [BL, PT, 6-month] × group [RBT + FES, RBT]). Bonferroni-adjusted pairwise comparisons identified time point differences. Chi-square tests of independence were used to compare the number of participants in each group who exceeded a clinically significant change (from BL to PT or BL to 6MFU) in each of the outcome measures for which MDC has been defined. Fall tracking data collected during the 6MFU period were reported descriptively, and Chi-square tests assessed group differences in fallers (ie, participants with ≥1 fall), frequent fallers (ie, participants with ≥2 falls), and falls resulting in injury. A relative risk ratio was determined based on the number of fallers in each group. The incidence rate of falls in each group were calculated and compared using Poisson regression. Time to first fall was analysed using Kaplan–Meier survival analysis. Analyses were conducted using R (version 4.1.2, R Foundation for Statistical Computing, Vienna, Austria) and alpha level was set to .05.

### Sample Size Justification

A sample size calculation based on the primary outcome was conducted in G*Power (version 3.0.10). The calculation was performed for a repeated measures ANOVA (within–between interaction), with a power level of 80%, alpha level of .05, and an anticipated effect size of 0.35. Ten participants per group were required. Assuming a 10% dropout rate, 22 participants in total were recruited.

## Results

### Participants

Twenty-two individuals with chronic motor iSCI participated in the study from April 2022 to December 2024. One participant withdrew after completing the BL assessments due to concerns over the travel demand. Another participant was withdrawn from the study due to poor attendance. Twenty participants (10 men and 10 women; 63.9 ± 14.8 years; 3.8 ± 2.8 years post-injury) were included in the analysis (Figure 2). The demographic and injury-related data and BL scores are presented in Table 1. All participants with the exception of 1 showed impaired reactive stepping ability on the baseline Lean-and-Release test. One participant in the RBT + FES group responded with single-step and 2-step responses to all 10 baseline Lean-and-Release trials. The assessor who administered the secondary outcomes remained blinded to group allocation for all participants. All 20 participants completed the fall tracking portion of the study during the 6 months following training. There were 3 adverse events reported: swelling at the ankle (n = 1), fall in the hospital lobby before a session with no injury sustained (n = 1), and mild skin irritation on the thighs from the harness straps (n = 1), which was corrected with additional padding.

**Figure 2.**
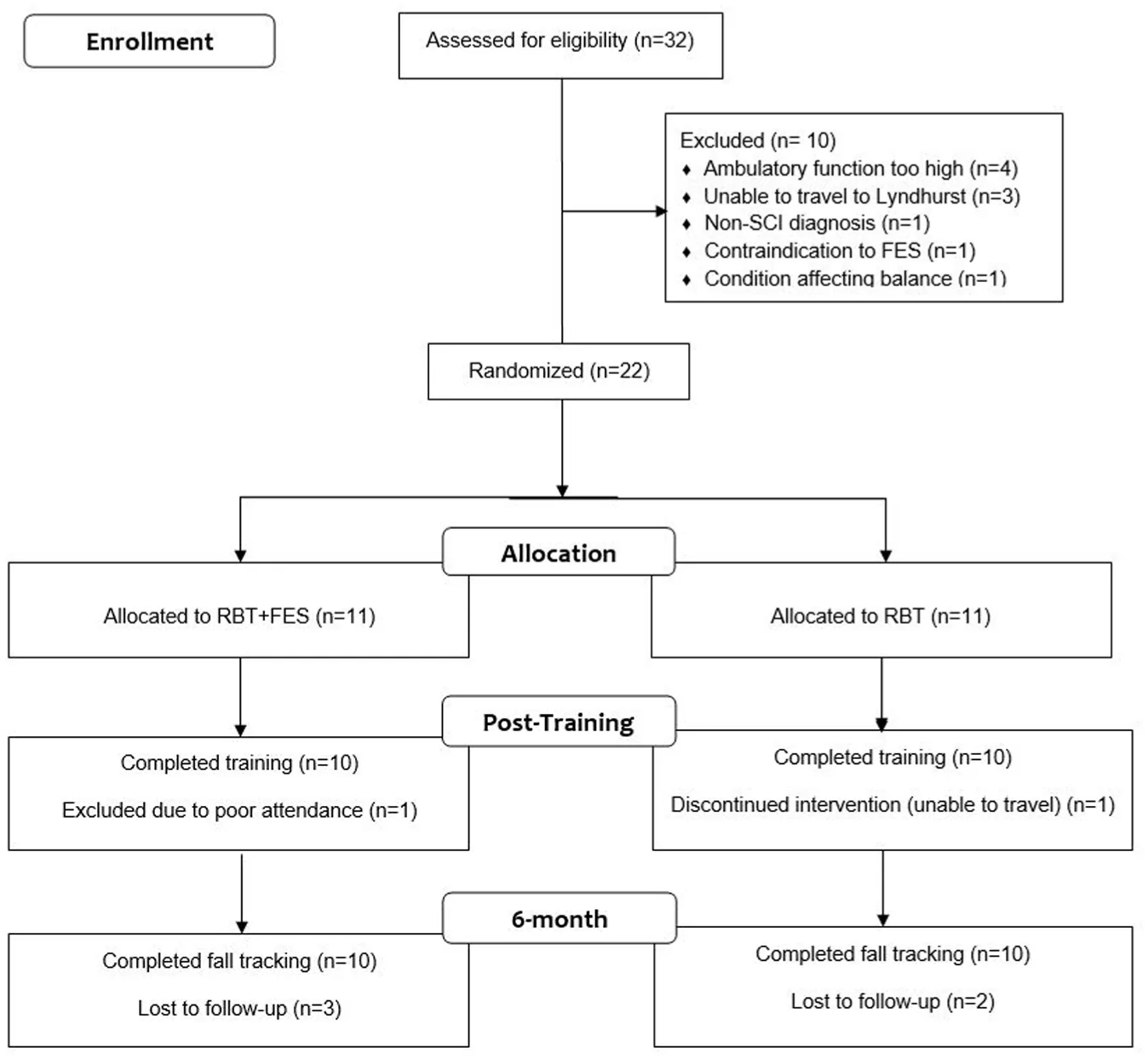
CONSORT flow diagram. Three participants in the RBT group were unable to complete the full array of MMT due to discomfort during positioning (ie, prone position laying on stomach). These participants were able to complete the MMT required for LEMS. One participant in the RBT group chose not to complete the personalised version of the ABC scale. The RBT group had 2 participants who chose not to complete the 6MFU assessments due to travel. Three participants from the RBT + FES group were unable to complete portions of the 6-month follow-up assessment including 2 participants who sustained injuries that prevented safe participation and 1 with scheduling constraints.

**Table 1.** Demographics, Injury-related Information, and Baseline Measures by Groups and Total Study Sample.

	Group		Total (n = 20)
Participant characteristics at baseline	RBT + FES (n = 10)	RBT (n = 10)	
Age (years)	65.4 (±11.7)	62.4 (±17.4)	63.9 (±14.8)
Gender			
Men	5	5	10
Women	5	5	10
Level of injury			
Cervical	7	7	14
Thoracic	2	3	5
Lumbar	1	0	1
Time since injury (years)	4.2 (±3.5)	3.4 (±1.7)	3.8 (±2.8)
Mechanism of injury			
Traumatic	5	5	10
Non-traumatic	5	5	10
Primary mobility aid used in daily life			
Wheelchair	2	1	3
Walker	4	5	9
Cane	4	4	8
L&R test: proportion of behavioural response			
Single-step	0.11 (±0.22)	0.08 (±0.14)	0.09 (±0.18)
2-Step	0.14 (±0.19)	0.20 (±0.21)	0.17 (±0.20)
≥3-Step	0.14 (±0.24)	0.12 (±0.11)	0.13 (±0.18)
Fall	0.62 (±0.32)	0.60 (±0.33)	0.61 (±0.32)
BBS			
(/56)	39.4 (±7.4)	39.1 (±12.9)	39.2 (±10.2)
Mini-BESTest			
Reactive Sub score (/6)	1.2 (±1.5)	1.4 (±1.2)	1.3 (±1.3)
MMT			
All^a^ (/90)	71.2 (±5.5)	67 (±9.5)	69.2 (±7.7)
LEMS (/50)	44.3 (±3.2)	41.3 (±6.6)	42.8 (±5.3)
Proprioception			
(/12)	11 (±1.7)	10 (±2.4)	10.5 (±2.1)
ABC Scale			
Original^b^ (%)	50.6 (±20.3)	55.2 (±19.4)	52.9 (±19.5)
Personalised (%)	20.1 (±16.9)	31.9 (±13.7)	25.7 (±16.2)
FES-I			
(/64)	41.2 (±12.4)	40.7 (±7.3)	41 (±9.9)

Abbreviations: L&R Test, lean-and-release test; BBS, Berg Balance Scale; Mini-BESTest, mini-Balance Evaluation Systems Test; MMT, manual muscle test; ABC Scale, Activities-specific Balance Confidence Scale; FES-I, Falls Efficacy Scale International.

a MMT grade summed for all 9 muscles tested bilaterally.

b Original ABC Scale (16 items). The proportion reported for the L&R Test is the mean proportion of trials classified as single-step, 2-step, ≥3 step, or a fall.

The 2 groups experienced a similar number of perturbations during training (RBT + FES: 47.3 ± 0.9, RBT: 47.3 ± 7.4, *t*_18_ = −0.027, *P* = .979). There were no significant between-group differences in the behavioural responses to those perturbations (single-step responses: 21.6 ± 6.6 for RBT + FES, 24.6 ± 7.2 for RBT, *t*_18_ = 0.984, *P* = .338; falls: 5.0 ± 5.6 for RBT + FES, 3.9 ± 3.3 for RBT, *t*_18_ = −0.540, *P* = .596).

### Primary Outcome: Lean-and-Release Test

Significant main effects of time were observed for the behavioural responses, including an increased proportion of single-step responses (*F*₂,₂₈ = 9.838, *P* < .001, η² = .144; Figure 3(A)) and a decreased proportion of falls (*F*₂,₂₈ = 13.023, *P* < .001, η² = .194; Figure 3(D)). No significant main effects of time were found for the proportion of 2-step (*P* = .579; Figure 3(B)) or ≥3-step responses (*P* = .310; Figure 3(C)). Post hoc tests demonstrated significant increases in proportion of single-step responses from BL to 6MFU (*P* = .023) and significant decreases in proportion of falls from BL to PT (*P* = .006) and BL to 6MFU (*P* = .022).

**Figure 3.**
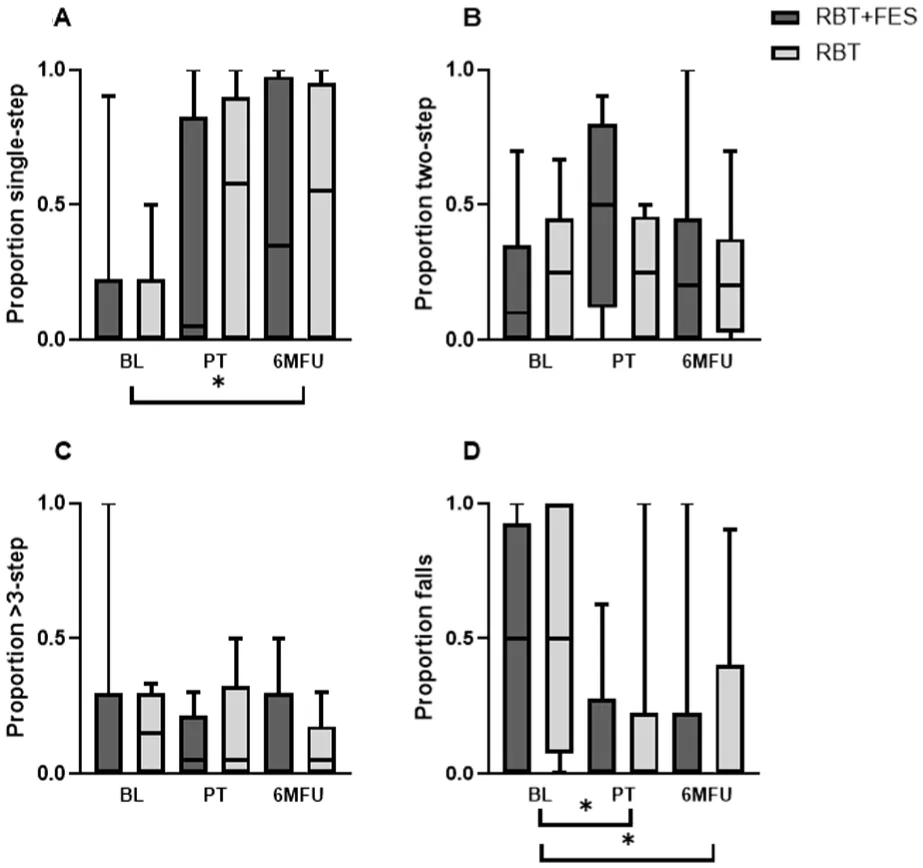
Boxplots of the behavioural responses for RBT + FES and RBT groups. (A) Proportions of single-step responses at baseline (BL), post-training (PT), and 6-month follow-up (6MFU). (B) Proportions of 2-step responses at BL, PT, and 6MFU. (C) Proportions of ≥3-step responses at BL, PT, and 6MFU. (D) Proportions of fall responses at BL, PT, and 6MFU. *Post hoc tests demonstrated significant increases in proportion of single-step responses from BL to 6MFU (*P* = .023) and significant decreases in proportion of falls from BL to PT (*P* = .006) and BL to 6MFU (*P* = .022).

No significant main effects of group were found for any behavioural response type (*P* > .376), indicating RBT + FES did not provide additional benefits over RBT alone. No significant group × time interactions were observed for any behavioural response type (*P* > .387).

There were no significant differences between groups in the proportion of participants who surpassed the threshold of 0.17 for change beyond the SEM (χ² = 0.000-0.808, *P* = .369-1.000). The median (IQR) changes in proportion of behavioural responses and number of participants exceeding the SEM for the 2 groups are reported in Table 2.

**Table 2.** Changes From Baseline to Post-training and Baseline to 6-month Follow-up Presented by Group.

	BL to PT		BL to 6MFU		Participants exceeding SEM/MDC**		Time effect	Group effect	Interaction effect
Outcome measure	RBT + FES	RBT	RBT + FES	RBT	RBT + FES	RBT	*P*-value	*P*-value	*P*-value
L&R test: proportion of behavioural response									
Single-step	0.000 (0.100)	0.400 (0.589)	0.000 (0.550)	0.350 (0.550)	3/10	6/10	<.001*	.819	.510
Two-step	0.400 (0.469)	0.000 (0.339)	0.100 (0.425)	−0.050 (0.492)	6/10	4/10	.579	.377	.388
≥3-step	0.000 (0.225)	−0.100 (0.450)	0.000 (0.225)	−0.067 (0.150)	2/10	3/10	.310	.642	.741
Fall	−0.388 (0.550)	−0.300 (0.575)	−0.150 (0.325)	−0.300 (0.450)	7/10	6/10	<.001*	.613	.708
BBS									
(/56)	6.300 (±3.335)	5.800 (±4.392)	6.125 (±4.518)	4.000 (±2.878)	6/10	4/10	<.001*	.652	.473
Mini-BESTest									
Reactive sub score (/6)	2.500 (±1.509)	2.700 (±1.947)	1.714 (±1.604)	2.250 (±1.753)	10/10	8/10	<.001*	.555	.338
ABC Scale									
Original^a^ (%)	17.375 (±7.659)	6.938 (±16.177)	10.313 (±6.505)	6.406 (±18.059)	6/10	3/10	<.001*	.927	.453
Personalised (%)	36.367 (±22.052)	30.639 (±15.667)	24.833 (±31.044)	26.036 (±21.226)	—	—	<.001*	.079	.994
FES-I									
(/64)	−10.000 (±8.219)	−6.300 (±7.945)	−7.222 (±7.328)	−9.625 (±9.410)	3/10	2/10	<.001*	.961	.605
MMT by Muscle Group (/10)									
Hip flexors	0.700 (±0.823)	0.300 (±0.483)	0.714 (±0.756)	0.625 (±1.061)	—	—	.059	.215	.903
Hip extensors	1.100 (±1.449)	1.143 (±1.215)	1.143 (±1.464)	0.833 (±1.329)	—	—	.120	.679	.950
Hip abductors	0.200 (±1.135)	0.500 (±1.414)	0.500 (±1.049)	0.167 (±1.329)	—	—	.186	.961	.787
Hip adductors	0.500 (±0.707)	1.000 (±0.926)	0.000 (±0.632)	0.833 (±1.329)	—	—	.064	.913	.644
Knee flexors	0.400 (±0.516)	0.143 (±0.378)	0.500 (±0.516)	0.167 (±0.983)	—	—	.115	.657	.882
Knee extensors	0.200 (±0.632)	0.100 (±1.287)	0.500 (±0.756)	0.500 (±0.926)	—	—	.068	.845	.997
Ankle dorsiflexors	0.500 (±0.707)	0.100 (±0.316)	0.375 (±0.518)	0.250 (±1.165)	—	—	.020*	.229	.860
Ankle plantarflexors	0.200 (±0.422)	0.100 (±0.876)	−0.125 (±0.641)	0.125 (±0.354)	—	—	.014*	.484	.981
Long toe extensors	0.400 (±1.350)	0.300 (±0.823)	0.125 (±1.642)	0.750 (±1.832)	—	—	.034*	.455	.769
Total MMT									
(/90)	4.200 (±3.615)	4.286 (±2.871)	4.333 (±3.615)	3.167 (±4.401)	—	—	<.001*	.349	.830

Abbreviations: BL, baseline; PT, post-training; 6MFU, 6-month follow-up; SEM, standard error of measure; MDC, minimal detectable change; L&R Test, Lean-and-Release Test; BBS, Berg Balance Scale; Mini-BESTest, mini-Balance Evaluation Systems Test; MMT, Manual Muscle Test; ABC Scale, Activities-specific Balance Confidence Scale; FES-I, Falls Efficacy Scale International.

Number of participants exceeding the SEM or MDC in each group. Results of 2-way mixed analysis of variances.

a Original ABC Scale (16 items).

b Number of participants in each group who exceeded SEM/MDC from BL to PT or BL to 6MFU.

* Significant result.

### Balance Measures

For BBS score, there was a significant main effect of time (*F*_2,28_ = 26.214, *P* < .001, η² = .111), but no significant main effect of group (*P* = .652) or group × time interaction (*P* = .473), suggesting standing balance control improved over time, with improvements similar for both groups (Figure 4(A)). Post hoc tests demonstrated no statistically significant differences in BBS scores between BL and PT (*P* = .108), BL and 6MFU (*P* = .083), or PT and 6MFU (*P* = 1.000).

**Figure 4.**
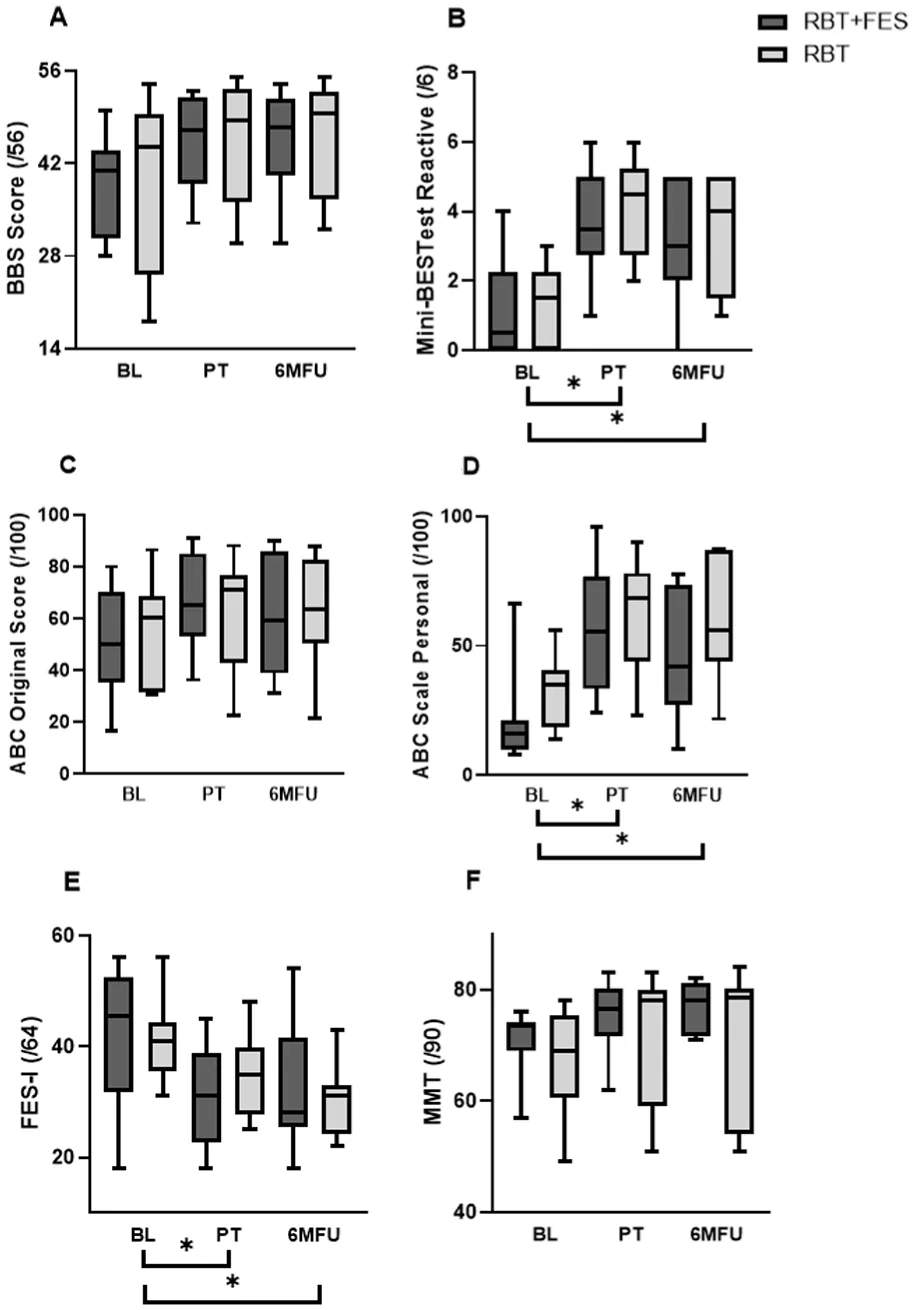
Boxplots of the balance, self-reported, and strength measures for the RBT + FES and RBT groups. (A) Boxplots of BBS scores at baseline (BL), post-training (PT), and 6-month follow-up (6MFU). (B) Boxplots of mini-BESTest: reactive sub-scores at BL, PT, and 6MFU. (C) Boxplots of original ABC scores at BL, PT, and 6MFU. (D) Boxplots of personalised ABC scores at BL, PT, and 6MFU. (E) Boxplots of FES-I scores at BL, PT, and 6MFU. (F) Boxplots of Total MMT scores at BL, PT, and 6MFU. *Post hoc tests demonstrated significant increases in mini-BESTest scores from BL to PT (*P* < .001) and BL to 6-month follow-up (*P* < .001), significant increases in personalised ABC scores from BL to PT (*P* < .001) and BL to 6MFU (*P* = .002), and significant decreases in FES-I scores from BL to PT (*P* = .021) and BL to 6MFU (*P* = .008).

For the mini-BESTest, there was a significant main effect of time (*F*_2,26_ = 23.995, *P* < .001, η² = .381), but no significant main effect of group (*P* = .555) or group × time interaction (*P* = .338), suggesting reactive balance control improved over time, with similar improvements in both groups (Figure 4(B)). Post hoc tests demonstrated significant increases in mini-BESTest scores from BL to PT (*P* < .001) and BL to 6MFU (*P* < .001), with no significant difference between PT and 6MFU (*P* = .835).

There were no significant differences between groups in the proportion of participants who surpassed the MDC for any balance measures (χ² = 0.000-0.556, *P* = .456-1.000). The mean ± SD changes and number of participants surpassing the MDC for both groups are reported in Table 2.

### Self-Reported Measures

Balance confidence, as measured by the original and personalised ABC scales, revealed a significant main effect of time (original: *F*_2,30_ = 13.460, *P* < .001, η² = .070; personalised: *F*_2,28_ = 23.236, *P* < .001, η² = .334), but no significant main effect of group (original: *P* = .927; personalised: *P* = .079) or group × time interaction (original: *P* = .453; personalised: *P* = .994), suggesting balance confidence improved over time similarly for both groups (Figure 4(C) and (D)). Post hoc tests demonstrated no significant differences in original ABC scores across time points (*P* > .180). In contrast, personalised ABC scores showed significant increases from BL to PT (*P* < .001) and BL to 6MFU (*P* = .002), with no significant change between PT and 6MFU (*P* = 1.000).

FES-I scores revealed a significant main effect of time (*F*_2,30_ = 11.980, *P* < .001, η² = .182), but no significant main effect of group (*P* = .961) or group × time interaction (*P* = .605), suggesting a similar reduction in fall concern over time for both groups (Figure 4(E)). Post hoc tests demonstrated FES-I scores significantly decreased from BL to PT (*P* = .021) and BL to 6MFU (*P* = .008), with no significant difference between PT and 6MFU (*P* = 1.000).

There were no significant differences between groups in the proportion of participants who surpassed the MDC for any self-reported measure (χ² = 0.000-0.808, *P* = .369-1.000). The mean ± SD changes and number of participants surpassing the MDC for both groups are reported in Table 2.

### Strength Measures

Total MMT score revealed a significant main effect of time (*F*_2,20_ = 11.297, *P* < .001, η² = .065), but no significant main effect of group (*P* = .349) or group × time interaction (*P* = .830), suggesting improvements in lower limb muscle strength over time that were similar across groups (Figure 4(F)). Post hoc tests demonstrated no significant differences in total MMT scores across time points (*P* > .400).

The following individual muscle groups showed a significant increase in MMT score over time: ankle dorsiflexors (*F*_2,36_ = 4.349, *P* = .020, η² = .096), ankle plantarflexors (*F*_2,36_ = 4.800, *P* = .014, η² = .147), and long toe extensors (*F*_2,36_ = 3.736, *P* = .034, η² = .083). Post hoc tests demonstrated no significant differences in MMT scores of the individual muscle groups across time points (*P* > .370). The hip and knee muscle groups did not demonstrate a significant effect of time (*P* > .058). There were also no significant group effects or group × time interactions for any of the specific muscle groups included in the MMT (*P* > .214). The mean ± SD changes in total MMT score and MMT scores of individual muscle groups are reported in Table 2.

For muscle groups with a MMT score of 4 or 5, peak force was measured with hand-held dynamometry. There were no significant group effects or group × time interactions for any of the muscle groups tested for peak force (*P* > .100). The mean peak force values of the following muscle groups showed a significant effect of time: right hip flexors (*F*_2,18_ = 5.057, *P* = .018, η² = .065), left and right knee extensors (left: *F*_2,24_ = 9.018, *P* = .001, η² = .045; right: *F*_2,24_ = 15.219, *P* < .001, η² = .140), left and right ankle dorsiflexors (left: *F*_2,24_ = 4.643, *P* = .020, η² = .026; right: *F*_2,24_ = 5.048, *P* = .015, η² = .024), and left and right ankle plantarflexors (left: *F*_2,24_ = 12.639, *P* < .001, η² = .048; right: *F*_2,24_ = 5.804, *P* = .009, η² = .032).

### Proprioception

Proprioception scores did not reveal any significant main effects or interactions (time: *P* = .429; group: *P* = .540; group × time interaction: *P* = .688), indicating no significant changes in proprioception over time or between groups.

### Follow-Up

During the 6MFU, 4 participants in each group experienced at least 1 fall. One participant in the RBT + FES group and 2 in the RBT group experienced 2 or more falls. There were no significant between-group differences in the proportion of fallers (χ² = 0.000, *P* = 1.000), frequent fallers (χ² = 0.000, *P* = 1.000), or falls resulting in injury (χ² = 0.000, *P* = 1.000). The relative risk of experiencing a fall in the RBT + FES group compared to the RBT group was 1.000 (CI: 0.342-2.926). The incidence rate of falls was not significantly different between groups (β = −.394, SE = 0.548, *P* = .472). Kaplan–Meier analysis revealed no significant difference in time to first fall between groups (χ² = 0.200, *P* = .960; Figure 5).

**Figure 5.**
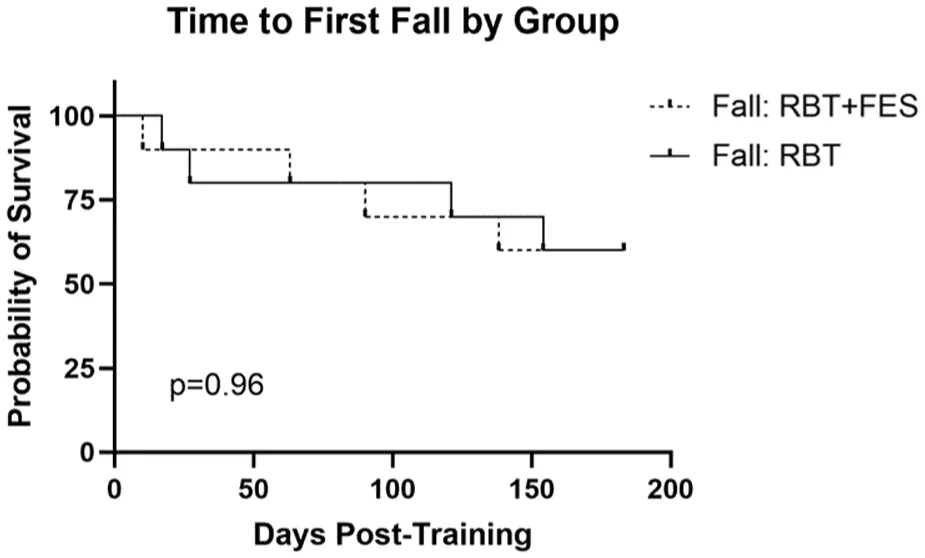
Kaplan–Meier curve of time to first fall for both groups.

## Discussion

Both RBT + FES and RBT alone led to significant improvements in reactive stepping ability, balance control, balance confidence, fall concern, and lower limb strength in individuals with motor iSCI. Contrary to our hypothesis, the addition of FES did not provide additional benefits over RBT alone. This study is among the first to demonstrate that FES can be integrated into RBT, a balance intervention that is increasingly recognised as an efficacious approach to improving balance control and reducing falls.^16-24^

In neurorehabilitation, FES can facilitate the repetitive practice of tasks that would otherwise not be possible due to muscle weakness.^28^ For people with iSCI, FES can have an orthotic effect that improves stepping kinematics, reduces compensatory strategies used during gait,^27,35^ and improves ankle stiffness during standing.^63^ RBT + FES was expected to have an orthotic effect that would increase the number of successful reactive steps executed, facilitating motor learning through repetitive practice.^64^ However, in a study completed in parallel with this trial, FES of the common fibular nerve was shown to have no orthotic effect on a lab-based assessment of the behavioural, spatial, or temporal parameters of reactive stepping for individuals with iSCI.^65^ Hence, it is possible that RBT + FES did not enhance the quality or quantity of reactive stepping practice beyond what was achieved with RBT alone. The absence of an immediate assistive benefit may provide a likely explanation for why FES did not provide added therapeutic effects in this trial. The physical therapist and kinesiologists delivering RBT + FES observed ankle eversion during the reactive steps of participants, which confirmed that the FES was active as ankle eversion is not a typical part of this movement pattern; however, whether the FES provided meaningful orthotic benefit during RBT is unknown. A future study that compares the quality and quantity of reactive steps taken during RBT sessions with and without FES would help address this question. Moreover, there may be alternative FES protocols that could facilitate an orthotic effect for reactive stepping. For example, stimulation to the quadriceps and gluteal muscles of the non-stepping leg may facilitate the speed and magnitude of body weight transfer^66,67^ required for a successful reactive step.

It was also expected that repetitive application of FES would increase muscle strength as FES can induce change in muscle properties (ie, increased cross-sectional area of type IIa oxidative muscle fibres).^68-73^ However, improvements in lower limb strength were similar between groups, suggesting no additional benefit of FES. The strength of the dorsiflexors and plantarflexors increased, which may account for the observed improvements in reactive stepping performance. These muscle groups are involved in the initiation and execution of a reactive step after a simulated forward fall.^12^ Therefore, it is likely that the repeated demand of perturbations during RBT led to an adaptation of increased strength in these muscles. A prior RBT study involving participants with iSCI reported no significant improvement in lower limb strength^16^; however, MMT alone was used to assess strength, which lacks sensitivity in the assessment of stronger muscle groups compared to hand-held dynamometry,^50^ and may explain the conflicting findings.

When electrical stimulation is delivered repeatedly, it can facilitate the reorganisation, formation and strengthening of neural connections.^33,74-76^ For example, repeated stimulation of the common fibular nerve increased lower limb motor evoked potentials in people with iSCI, suggesting an increase in corticospinal activity.^33^ RBT + FES may have facilitated neuroplasticity as the peripheral input from stimulation occurs simultaneously with increased cortical activation brought on by participation in challenging, task-specific balance tasks (ie, Hebbian mechanism).^75,76^ However, the extent of cortical engagement required for reactive stepping may not be sufficient to drive meaningful plasticity via Hebbian mechanisms. Reactive stepping is largely governed by long-loop reflexes and long-latency pathways, with cortical involvement being indirect and primarily mediatory rather than initiatory.^77,78^ This differs from more voluntary tasks, where corticospinal engagement is direct and substantial, making peripheral stimulation more likely to strengthen the relevant pathways. There are subcortical descending pathways (ie, reticulospinal and vestibulospinal tracts) that play key roles in locomotion, postural control, and reactive stepping^79-81^; however, the effect of FES on these pathways is unknown. The lack of added benefit from FES found in this study may therefore suggest that strengthening of neural pathways through peripheral stimulation is more effective in tasks with a greater reliance on cortical control, or that intensive, repetitive, and challenging task-specific training on its own may be sufficient for supporting neuroplasticity and functional change.^64^ Spinal stimulation is an emerging neuromodulatory approach that may be a better match for a behaviour like reactive stepping since this type of electrical stimulation targets short-latency posterior root-muscle reflexes and longer-latency reflexes that are likely polysynaptic in nature.^82^ The findings reported here align with recent studies suggesting that FES combined with task-specific training does not result in greater functional outcomes compared to task-specific training alone for people with iSCI.^83-86^

Our study contributes a more thorough understanding of the effects of interventions that directly target reactive postural control, which is an understudied area for the iSCI population.^14^ Most clinical trials focussing on balance outcomes after iSCI have involved walking interventions or upright balance training with visual feedback.^14^ One review identified the combination of FES with body weight supported training as a promising approach for improving balance in people with iSCI.^87^ Another understudied area in iSCI rehabilitation is the impact of balance training on fall incidence.^14^ While our study found that 40% of participants fell in the 6 months following training, it is unclear whether this incidence is an improvement as the occurrence of falls was not tracked pre-training.

Compared to the RBT group, more participants in the RBT + FES group surpassed the MDC for the secondary outcome measures (see Table 2). Notably, all 10 participants in the RBT + FES group surpassed the MDC of the mini-BESTest reactive subscale while 8/10 RBT participants did so. Compared to the RBT group, the RBT + FES group also had twice as many participants exceed the MDC of 15% on the ABC scale, a measure of balance confidence.

### Limitations

The study has several limitations worth noting. First, convenience sampling combined with broad inclusion criteria may have affected the results, as the majority of participants were high functioning and likely did not need FES to facilitate their participation in RBT. Second, the sample size was small, and it is possible that some secondary analyses were not adequately powered. Third, the falls data depended on participants remembering to report a fall in a timely manner. It is possible that some falls were not reported.

## Conclusions

Both RBT and RBT + FES are effective interventions for improving reactive stepping ability, balance control, balance confidence, fall concern, and lower limb strength in individuals with chronic motor iSCI. However, the FES protocol applied in this study did not provide additional benefits over RBT alone.

## Supplemental Material

sj-xlsx-1-nnr-10.1177_15459683261454942 – Supplemental material for Functional Electrical Stimulation Combined With Reactive Balance Training for Individuals With Incomplete Spinal Cord Injury: A Randomised Clinical Trial Supplemental material, sj-xlsx-1-nnr-10.1177_15459683261454942 for Functional Electrical Stimulation Combined With Reactive Balance Training for Individuals With Incomplete Spinal Cord Injury: A Randomised Clinical Trial by Matthew G. Heffernan, Katherine Chan, Jonguk Lee, Lovisa Cheung, Elizabeth L. Inness, Kei Masani and Kristin E. Musselman in Neurorehabilitation and Neural Repair
